# Application of Generative Artificial Intelligence Models for Accurate Prescription Label Identification and Information Retrieval for the Elderly in Northern East of Thailand

**DOI:** 10.3390/jimaging11010011

**Published:** 2025-01-06

**Authors:** Parinya Thetbanthad, Benjaporn Sathanarugsawait, Prasong Praneetpolgrang

**Affiliations:** School of Information Technology, Sripatum University, Bangkok 10900, Thailand; parinya.the@spumail.net (P.T.); benjaporn.sa@spu.ac.th (B.S.)

**Keywords:** AI in healthcare, prescription label identification, visual question answering, medical image understanding, LLM in healthcare

## Abstract

This study introduces a novel AI-driven approach to support elderly patients in Thailand with medication management, focusing on accurate drug label interpretation. Two model architectures were explored: a Two-Stage Optical Character Recognition (OCR) and Large Language Model (LLM) pipeline combining EasyOCR with Qwen2-72b-instruct and a Uni-Stage Visual Question Answering (VQA) model using Qwen2-72b-VL. Both models operated in a zero-shot capacity, utilizing Retrieval-Augmented Generation (RAG) with DrugBank references to ensure contextual relevance and accuracy. Performance was evaluated on a dataset of 100 diverse prescription labels from Thai healthcare facilities, using RAG Assessment (RAGAs) metrics to assess Context Recall, Factual Correctness, Faithfulness, and Semantic Similarity. The Two-Stage model achieved high accuracy (94%) and strong RAGAs scores, particularly in Context Recall (0.88) and Semantic Similarity (0.91), making it well-suited for complex medication instructions. In contrast, the Uni-Stage model delivered faster response times, making it practical for high-volume environments such as pharmacies. This study demonstrates the potential of zero-shot AI models in addressing medication management challenges for the elderly by providing clear, accurate, and contextually relevant label interpretations. The findings underscore the adaptability of AI in healthcare, balancing accuracy and efficiency to meet various real-world needs.

## 1. Introduction

Thailand is experiencing a rapid shift toward an aging society, with significant implications for healthcare as older adults require specialized support for managing complex health conditions. By June 2023, individuals aged 65 and older accounted for over 19.4% of the population, and projections suggest this demographic will grow to 25% by 2028 [1,2]. This accelerated aging trend presents challenges for the healthcare system, as elderly individuals face heightened risks for chronic conditions and complex medication regimens [3]. Unlike other nations that have transitioned gradually, Thailand’s swift demographic change is creating an urgent need for adaptive healthcare solutions, especially in medication management—a vital component of elderly care.

Chronic diseases such as hypertension, diabetes, and cardiovascular conditions are prevalent among older adults and often co-occur, necessitating multiple medications [4]. This phenomenon, known as multi-morbidity, complicates healthcare delivery and medication management, increasing the risk of drug interactions and adverse effects. Managing multiple medications requires not only medical oversight but also lifestyle adjustments, dietary modifications, and frequent health monitoring. The psychological toll of managing chronic diseases—potentially leading to anxiety or depression—further complicates adherence, making a holistic approach to elderly care essential [4].

Effective medication management is critical for preserving health and preventing complications that could lead to hospitalization or worse. The complex regimens common in elderly care elevate the risk of medication errors, a significant cause of preventable harm [5,6]. Inadequate medication management impacts individual health and places additional strain on healthcare systems. Optimal medication management requires clear communication with healthcare providers, regular medication reviews, and practical adherence strategies. In an aging society, addressing these challenges with innovative solutions becomes crucial for enhancing the quality of life and health outcomes for older adults while also alleviating pressures on healthcare resources.

For elderly patients, medication management encompasses a range of intricate tasks that become more challenging with age. These tasks include recognizing multiple medications, understanding complex dosing schedules, monitoring for side effects, and adapting to evolving medication regimens. Visual impairments, cognitive decline, and reduced dexterity further complicate these tasks, often leading to medication errors that can jeopardize patient safety [7]. These challenges highlight the importance of developing support systems tailored to the specific needs of elderly patients, empowering them to safely manage their medications in daily life.

Age-related cognitive and physical impairments exacerbate medication management difficulties. Issues such as presbyopia make reading small print on labels challenging, while cognitive decline makes it difficult to follow dosing schedules accurately [8,9]. Conditions like arthritis reduce manual dexterity, adding further obstacles to handling medications. This complex interplay of physical and cognitive declines increases the risk of mismanagement, positioning AI-powered tools as promising solutions to support elderly individuals and mitigate the impact of cognitive decline on medication adherence.

For family members and informal caregivers, managing medications for elderly loved ones can be overwhelming, often due to a lack of medical training or expertise. Caregivers may be responsible for administering multiple medications with varied schedules, which can lead to errors that endanger the patient’s health [10]. The added stress and potential for caregiver burnout underline the need for reliable tools to simplify medication management, benefiting both caregivers and patients. Such tools should focus on facilitating accurate medication administration through user-friendly design and enhanced accessibility.

Recent advances in artificial intelligence (AI) applications within healthcare demonstrate transformative potential for medication management. From disease prediction to diagnostic imaging, AI technologies like Optical Character Recognition (OCR) and Large Language Models (LLMs) are evolving to provide effective support [11,12,13,14]. Combining OCR technology with LLMs or visual LLMs offers a novel way to assist elderly patients in reading and understanding medication labels, especially in scenarios where label clarity or text recognition may be inconsistent. OCR, particularly for Thai text, presents unique challenges, but the integration of LLMs can help refine and validate recognized text, enhancing accuracy and reliability [15,16].

This study introduces two AI-driven approaches tailored to the unique medication management needs of elderly individuals in Thailand. The first is a Two-Stage model that pairs EasyOCR [17] for text extraction with Qwen2-72b-instruct [18], an advanced LLM for interpreting and contextualizing medication information. The second is a Uni-Stage Visual Question Answering (VQA) model, Qwen2-72b-VL [18], which processes both visual and textual data in a single step to offer efficient, real-time responses. Both approaches integrate Retrieval-Augmented Generation (RAG) [19] with a curated set of drug images from DrugBank [20], providing reliable, context-sensitive support for drug identification and usage. To thoroughly evaluate these approaches, we employ the Retrieval Augmented Generation Assessment (RAGAs) framework [21], focusing on metrics including accuracy, precision, recall, F1-score, LLM Context Recall, Factual Correctness, Faithfulness, and Semantic Similarity. To offer a comprehensive understanding of our contributions, the remainder of this paper is organized as follows:

1. Section 2 (Literature Reviews) surveys existing research on drug label identification and Large Language Models, highlighting key limitations and knowledge gaps.
2. Section 3 (Methodology) outlines our model architectures, data collection strategies, and the integration of RAG.
3. Section 4 (Results) presents both quantitative and qualitative evaluations of the proposed models using the RAGAs framework.
4. Section 5 (Discussion) interprets the findings in relation to previous research, exploring broader implications and addressing potential limitations.
5. Section 6 (Conclusion and Future Work) summarizes key insights and proposes directions for extending the system to wider clinical and home healthcare settings.

## 2. Literature Reviews

### 2.1. Related Works

Significant progress has been made in drug label identification through machine learning, enhancing the recognition and interpretation of complex pharmaceutical information. Liu et al. (2020) proposed the Drug Label Identification through Image and Text Embedding (DLI-IT) model, utilizing a dataset from Daily-Med with 4114 opioid and non-opioid drug images, along with 300 additional test images [22]. This model combines a Connectionist Text Proposal Network (CTPN) with a deep learning VGG-16 architecture and Long Short-Term Memory networks, accurately localizing text within drug label images [23,24,25]. Tesseract OCR was employed for text recognition, capturing essential drug names and label information [26]. DLI-IT achieved 88% accuracy when combining image and text features, underscoring the advantages of multimodal data integration for improved label recognition [22].

In a similar study, Ting et al. (2020) developed a Deep Learning Drug Identification (DLDI) model using YOLOv2 to identify drugs on blister packaging [27]. With a dataset of 36,000 images capturing both front and back views of blister packs from outpatient departments in Taiwan, DLDI achieved 96.26% accuracy on the back side due to distinct color and textural features. However, front-view misidentifications, especially with visually similar drugs like Ritalin and Atenolol, highlighted the need for robust feature extraction methods to minimize confusion in drug package recognition [28].

In 2022, Gromova and Elangovan introduced a model for cylindrical drug bottles, leveraging a Deep Convolutional Neural Network (DCNN) to recognize key points on the bottle’s curved surface [29]. Using a cylindrical warp model, this approach transformed the label’s curved surface into a flat representation, enabling accurate label content analysis. Canny edge detection and Hough transforms identified edges and geometries [30], while EfficientNet and MobileNet architectures ensured robust performance under varied lighting conditions [31,32]. With 98% accuracy in locating label points, this method demonstrated potential for enhancing drug safety by capturing critical information on cylindrical packaging.

More recently, You and Lin (2023) introduced a Two-Stage induced deep learning (TSIDL) model that categorizes drug packaging with high precision. This model applies a hierarchical classification approach, initially categorizing drugs broadly and then refining classifications with induced learning. With a dataset of 108 diverse packaging types, the model achieved 99.39% accuracy, benefiting from data augmentation techniques like rotation and scaling [33]. The high precision and rapid inference time highlight TSIDL’s suitability for real-time applications, particularly in high-throughput environments like pharmacies.

While these traditional deep learning models demonstrate effectiveness, they face limitations when handling the nuanced complexities of pharmaceutical labels. These challenges include difficulties with diverse fonts, color schemes, and layouts essential for distinguishing similar drugs [22,28,29,33,34,35]. Furthermore, such models often require extensive, specifically-tuned datasets, creating scalability issues. Fine-tuning for new label variations also risks overfitting, reducing generalizability—particularly problematic in dynamic pharmaceutical environments where label designs frequently change. As summarized in Table 1, despite achieving high accuracy, each previous study faced specific limitations, underscoring the need for more robust and adaptable solutions.

**Table 1:** Summary of previous works on drug label identification models.

Study	Methodology	Dataset	Accuracy	Limitations
Liu et al. (2020) [22]	DLI-IT (CTPN + VGG-16 + LSTM)	Daily-Med (4114 images)	88%	Requires multimodal data integration; limits with text complexity
Ting et al. (2020) [28]	YOLOv2 (Blister Packaging)	36,000 images	96.26%	Front-view limitations; similar packaging leads to errors
Gromova and Elangovan (2022) [29]	DCNN (Cylindrical Bottles)	Custom, various lighting	98%	Handling curved surfaces only; lighting sensitivity
You and Lin (2023) [33]	TSIDL (Two-Stage Induced)	108 packaging types	99.39%	Focus on speed; limited contextual diversity

Recent advancements in Generative AI and LLM with VQA capabilities offer promising solutions [36,37,38,39,40]. These models provide zero-shot learning capabilities, allowing them to interpret and analyze new drug labels without retraining, reducing dependency on large labeled datasets, and mitigating traditional models’ limitations with novel inputs. Incorporating RAG enables LLM and VQA models to further improve label interpretation by cross-referencing curated datasets like DrugBank, yielding more reliable and contextually accurate outputs. This integration supports real-time responses and context-driven interpretations critical for elderly users to understand dosage instructions, usage, and potential interactions.

To evaluate these advanced systems, RAGAs metrics offer a nuanced framework for assessing LLM performance across critical factors, including LLM Context Recall, Factual Correctness, Faithfulness, and Semantic Similarity. By emphasizing both accuracy and contextual alignment, RAGAs metrics ensure drug identification systems generate outputs tailored to user needs. This approach is particularly beneficial in elderly care, where clear and accurate medication information is essential for safety and adherence.

Integrating these advanced models into healthcare involves challenges related to interpretability, reliability, and validation. Ensuring model outputs are clear and verifiable remains critical for clinical applications. Addressing these issues requires ongoing research to enhance transparency and build trust in AI-assisted tools. Despite these challenges, applying Generative AI, LLMs, and VQA technologies to pharmaceutical labeling represents a transformative approach, improving safety, accessibility, and efficiency in drug identification and management.

### 2.2. Large Language Models

LLMs, designed to process extensive text datasets, enable human-like responses and complex language understanding. Beyond basic text processing, LLMs excel in nuanced classification, information extraction, and answering complex queries, making them highly valuable in healthcare for tasks such as interpreting medical literature and supporting clinical decision-making. However, their integration into sensitive domains like healthcare presents challenges such as ensuring patient privacy and mitigating biases. Additionally, the need for domain-specific accuracy adds complexity to LLM deployment, as they must reliably interpret specialized terminology and context-specific information. Recent advancements have expanded LLMs’ multilingual and multimodal capabilities, increasing their potential reach in global healthcare initiatives. For instance, models like XLM-RoBERTa and mT5 demonstrate strong cross-linguistic performance, essential for interpreting pharmaceutical labels in diverse languages and regions [37,38,41,42].

The field has progressed rapidly, marked by models such as Google’s PaLM 2, which showcases advanced multilingual capabilities and excels at complex reasoning tasks. BLOOM, developed by the BigScience initiative, represents a collaborative effort toward open, multilingual models optimized for diverse research domains. Meanwhile, Mistral AI’s compact Mistral 7B model is designed for resource-constrained environments, delivering high performance despite its smaller size. Each model’s unique architecture and capabilities enable researchers to address various challenges in healthcare [43,44,45].

A notable advancement in LLM technology is OpenAI’s GPT-4o, recognized for its enhanced contextual understanding, factual accuracy, and instruction-following abilities. These attributes make GPT-4o valuable in healthcare, where precise language comprehension is critical. For instance, in public health contexts, GPT-4o has demonstrated strong performance in epidemiological data analysis and policy formulation. However, its closed-source nature limits transparency, essential for researchers needing insight into decision-making processes, especially in sensitive healthcare applications [46].

In healthcare, specialized LLMs such as PubMedBERT and BioBERT, trained on biomedical literature, enhance understanding of medical terminology and clinical language. Advanced models like Med-PaLM 2 and BioGPT extend these capabilities to complex medical question answering and information retrieval, assisting in tasks like research summarization, case study interpretation, and preliminary diagnostics. While these models provide powerful tools for healthcare professionals, their integration into workflows must complement, not replace, expert judgment [47,48].

For this study, we utilize Qwen2-7b-instruct alongside OCR to aid in prescription label identification. OCR extracts text from medication labels, and Qwen2-7b-instruct processes this text to generate clear, contextually relevant information for elderly patients and caregivers. Unlike general-purpose models like GPT-4o, Qwen2-7b-instruct excels in instruction-following tasks and is well-suited for the structured outputs required in healthcare. Incorporating Retrieval-Augmented Generation (RAG) allows the LLM to reference a curated drug dataset, such as DrugBank, ensuring fact-checked and contextually accurate responses. This approach is particularly beneficial in elderly care, where reliable medication identification can reduce errors and support safer health outcomes.

### 2.3. Visual Question Answering Model

VQA is a multimodal AI task that combines computer vision and language processing to answer questions based on image content [39]. In healthcare, VQA models enable systems to interpret complex visual information, such as prescription labels, while answering natural language queries—an increasingly relevant capability for medication management. Traditional VQA models relied on separate encoders for text and image inputs, merging these representations to predict answers. Recent models have adopted transformer-based architectures, achieving high performance across natural language and visual tasks. State-of-the-art VQA models now utilize pre-trained vision–language models and unified transformers, significantly enhancing their ability to handle diverse queries and interpret complex images [39].

Training VQA models typically involves large datasets of image question–answer pairs, such as VQA v2, GQA, and CLEVR [49,50]. In medical domains, specialized datasets like VQA-RAD and PathVQA address unique healthcare challenges, such as interpreting radiology images or pathology slides [51,52]. Training strategies include supervised learning, contrastive learning, and self-supervised pre-training, enhancing the model’s ability to generalize across diverse visual inputs. Despite these advancements, challenges persist in ensuring robustness to visual variations, reasoning capabilities, and compositional generalization, such as interpreting novel label formats or unconventional packaging layouts.

Models such as MiniCPM and its visual variant, MiniCPM-LLaMa3-V 2.5, show strong results in tasks requiring language and visual comprehension [36]. These models excel in OCR and instruction-following tasks, making them highly relevant for prescription label identification. Their efficient architecture and multimodal capabilities enable deployment in resource-constrained environments where lightweight models are crucial [36].

The selection of Qwen2-72b-VL for prescription identification is strategic, given its multimodal capabilities [18]. Qwen2-72b-VL seamlessly processes visual information from prescription images alongside textual queries, addressing challenges like interpreting diverse label layouts and ensuring contextual relevance. Its high OCR accuracy supports reliable text extraction from complex labels, while its training on instruction-following datasets enables flexible, intuitive querying. For example, the model can assist pharmacists or elderly users by answering natural language queries like, “What is the dosage for this medication?” or “Are there any storage requirements?” in real time. Although not explicitly trained on medical data, its exposure to diverse image–text pairs positions it well for tasks involving prescription labels and medication management.

Our decision to integrate EasyOCR with Qwen2-72b-instruct is guided by their complementary strengths. EasyOCR offers robust performance in Thai text recognition—an essential requirement given the unique linguistic complexities of Thai prescription labels, including multiple fonts, diacritical marks, and varied orientations. Many open-source OCR solutions underperform in handling such intricate scripts, while EasyOCR consistently demonstrates competitive accuracy under real-world conditions (e.g., suboptimal lighting, varying camera angles) [17].

Meanwhile, Qwen2-72b-instruct stands out among LLMs for its strong instruction-following capabilities, enabling it to produce consistent, structured outputs that are crucial in healthcare contexts. Its capacity for high contextual comprehension ensures that extracted text from EasyOCR is transformed into patient-friendly instructions, dosage guidelines, and cautionary statements. Moreover, Qwen2-72b-instruct’s multilingual capabilities bolster its utility in diverse healthcare environments where overlapping languages and transliterated content may appear on drug packaging. By combining these two models—one specialized in text extraction and the other in interpretative, instruction-focused language tasks—our integrated system addresses the limitations of previous deep learning methods, providing accurate label recognition and meaningful, context-rich medication guidance for elderly patients [18].

## 3. Methodology

This study employs a comprehensive approach that integrates Optical Character Recognition (OCR), Large Language Models (LLMs), and Retrieval-Augmented Generation (RAG) to enhance the accuracy and contextual relevance of prescription label identification for elderly patients in Thailand. This methodology addresses the challenges elderly users face in medication management by providing clear, accurate, and accessible explanations of drug labels.

### 3.1. Model Architecture and Approach

To achieve accurate and comprehensive drug label interpretation, we implement two distinct models:

#### 3.1.1. Two-Stage OCR + LLM Model

This model combines EasyOCR [17] with the Qwen2-72b-instruct LLM [18] in a two-step, sequential process designed to ensure high accuracy and context-driven interpretation:

- OCR Stage: EasyOCR is employed to extract text from images of prescription labels. This OCR tool is particularly suited to handle the diverse font styles, sizes, orientations, and Thai language characters commonly found on pharmaceutical labels within Thailand.
- LLM Interpretation Stage: The extracted text is processed by Qwen2-72b-instruct, a language model selected for its strong instruction-following capabilities. This stage leverages the model’s contextual understanding to generate user-friendly interpretations, enriched with predefined knowledge for clarity and precision.

#### 3.1.2. Uni-Stage VQA Model

In this approach, we use Qwen2-72b-VL [18], a model equipped to interpret visual and textual data in a single step, streamlining the process:

- Direct Image Processing: The VQA model processes the entire image of the prescription label directly, bypassing the need for a separate OCR stage.
- Simultaneous Visual and Textual Interpretation: This model interprets visual cues and textual data concurrently, providing real-time responses to specific questions about the label. This approach is particularly advantageous in scenarios that require rapid analysis and response.

### 3.2. RAG and Knowledge Integration

Both models integrate RAG to enhance their contextual understanding and factual accuracy. A curated dataset of our collected prescription names sourced from DrugBank provides domain-specific knowledge, enabling the models to accurately interpret drug names, dosages, usage instructions, and potential warnings [20]. By referencing this dataset, RAG allows the LLM to verify and contextualize label information, ensuring outputs that are both factually reliable and contextually relevant for elderly patients who may have difficulty navigating complex medication instructions [19].

### 3.3. Evaluation Metrics Using RAGAs

To assess the effectiveness of the models, we employ the RAGAs evaluation framework, which offers metrics critical to healthcare applications:

- Accuracy, precision, recall, and F1-score: these are standard classification metrics that gauge how well the system can identify and interpret correct label information.
- LLM Context Recall: This metric measures the model’s ability to retrieve and retain contextually relevant information from external knowledge sources (e.g., DrugBank) to ensure responses are both informative and tailored to specific drug labels. For this metric, ground truth answers are referenced to validate that the essential information required for a complete response is present.
- Factual Correctness: This metric assesses the factual accuracy of the model’s responses, verifying that the generated information is consistent with verified pharmaceutical data. High Factual Correctness is vital in healthcare contexts to minimize the risk of misinformation, particularly for medication instructions.
- Faithfulness: This metric evaluates the extent to which model-generated responses are faithful to the information extracted from drug labels and context sources, minimizing deviation from the source content. Maintaining Faithfulness ensures that the model delivers responses that accurately reflect label data, which is critical in healthcare to prevent potentially harmful misunderstandings.
- Semantic Similarity: This metric measures the semantic alignment of the model’s outputs with expert-verified explanations, focusing on producing responses that closely match the language and terminology healthcare professionals would use. High Semantic Similarity ensures that the model’s outputs are both understandable and contextually relevant, fostering trustworthiness for elderly patients and caregivers.

These metrics provide a comprehensive evaluation of each model’s ability to generate accurate, contextually relevant, and user-friendly outputs, all of which are essential in supporting elderly patients and caregivers in effective medication management [21].

### 3.4. Data Collection and Preprocessing

Our data collection strategy was designed to capture the diversity of prescription labels used across Thailand’s healthcare system. We gathered 100 unique prescription labels from various public healthcare facilities, ensuring representation of different medication types, label formats, and instructions, as summarized in Table 2. This dataset includes essential information such as medication names and dosage instructions, all of which are vital for patient safety.

**Figure 1:**
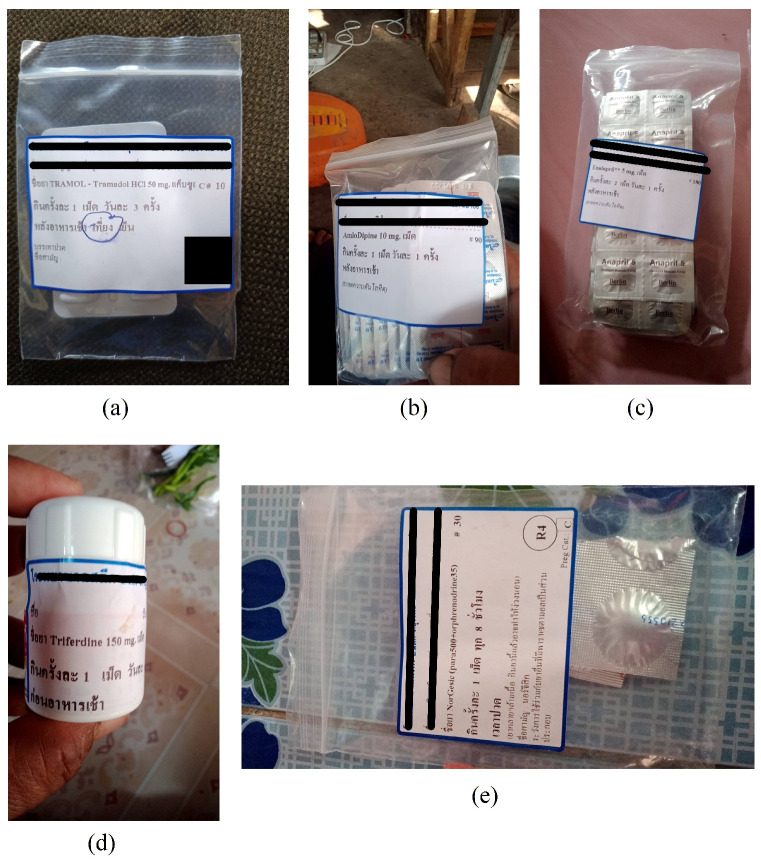
Example of prescription images with Thai instructions. (**a**) TRAMOL—Tramadol HCl 50 mg: “take 1 tablet, 3 times a day, after meals”. (**b**) Amlodipine 10 mg: “take 1 tablet, once a day, after breakfast.” (**c**) Enalapril 5 mg: “take 2 tablets, once a day, after breakfast.” (**d**) Triferdine 150 mg: “take 1 tablet, once a day, before breakfast.” (**e**) Norgesic (Paracetamol 500 mg + Orphenadrine 35 mg): “take 1 tablet when in pain. This is a muscle relaxant and may cause drowsiness”. Other Thai text includes drug and generic names.

To simulate real-world conditions, the labels were captured under varying lighting conditions, angles, and resolutions using different mobile devices. This approach ensures the models’ robustness across realistic usage scenarios, thereby improving reliability in diverse situations that elderly patients may encounter daily. In compliance with the Thai Personal Data Protection Act (PDPA), all personal identifiers were anonymized to ensure privacy and adherence to ethical standards (see Figure 1).

**Figure 2:**
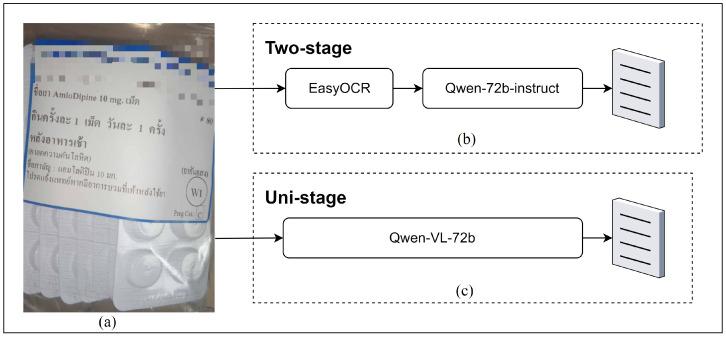
Workflow of Two-Stage and Uni-Stage models for drug label interpretation. (**a**) Input label (Amlodipine 10 mg) with Thai instructions, e.g., “take 1 tablet once a day, after breakfast”. (**b**) Two-stage model: EasyOCR extracts text, then Qwen-72b-instruct interprets it. (**c**) Uni-stage model: Qwen-VL-72b directly processes the image.

Figure 2 illustrates the workflow for this comparative study, detailing the processes involved in both the Two-Stage (OCR + LLM) and Uni-Stage (VQA) models. This comparison facilitates a comprehensive understanding of each model’s strengths and limitations, providing guidance for future development of robust, versatile, and contextually aware language models in healthcare.

### 3.5. Prompts and Testing

**Figure 3:**
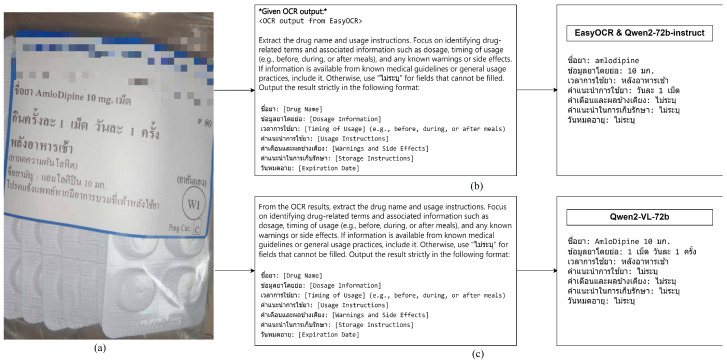
Comparison of Two-Stage and Uni-Stage model outputs for drug label interpretation. (**a**) Input image of a sample drug label (Amlodipine 10 mg). (**b**) The Two-Stage model (EasyOCR followed by Qwen2-72b-instruct) output: Drug Name: amlodipine; Dosage Information: 10 mg; Timing of Usage: after breakfast; Usage Instructions: once a day; Warnings and Side Effects: Not specified; Storage Instructions: Not specified; Expiration Date: Not specified. (**c**) The Uni-Stage model (Qwen2-VL-72b) output: Drug Name: Amlodipine 10 mg; Dosage Information: 1 tablet once a day; Timing of Usage: after breakfast; Usage Instructions: Not specified; Warnings and Side Effects: Not specified; Storage Instructions: Not specified; Expiration Date: Not specified.

To rigorously evaluate the LLM and VQA models, we developed a set of real-world prompts to assess each model’s ability to interpret and explain information for elderly users accurately. These prompts simulate practical scenarios and address common questions related to dosage, administration, and safety instructions. This testing approach provides insights into the strengths and weaknesses of each model, laying the groundwork for future improvements in AI healthcare support systems (see Figure 3).

### 3.6. Hyperparameter Configuration

The implementation of our integrated system necessitates the careful selection of hyperparameters that govern the generation and interpretation behaviors. While some research paradigms within the field of AI fine-tune these parameters extensively, our study adopts standard or widely accepted default settings. This strategy aims to achieve a balance between model performance (encompassing metrics such as accuracy and Faithfulness) and the critical requirements of consistency and reproducibility. The primary hyperparameters employed for both the Two-Stage and Uni-Stage approaches are detailed below:

- The LLM’s and VQA’s temperature parameter is configured to 0.0 during the inference phase. This conservative setting promotes deterministic and focused output generation, as opposed to more creative or diverse responses. In the context of healthcare, where factual accuracy and consistency are paramount, introducing randomness through a higher temperature is deemed undesirable. Maintaining a temperature of 0.0 effectively mitigates the risk of generating hallucinated or speculative content.
- For text generation, we utilize standard default configurations for top-*k* sampling and nucleus sampling (top-*p*). Specifically, top-*k* is disabled by setting k=0, and top-*p* is set to 1.0. This configuration compels the decoder to produce the most probable next token at each timestep without applying stochastic filtering. These deterministic settings are particularly crucial when generating medication-related advice, where precision is essential.
- To prevent overly lengthy or tangential responses, the maximum number of generated tokens is capped at 128. This limit is deemed sufficient to convey standard prescription or dosage instructions concisely, thereby enhancing overall clarity, particularly for elderly users.
- The repetition penalty is set to its default value of 1.0. This parameter is designed to mitigate the occurrence of repeated phrases during generation. Our empirical observations indicated that this parameter did not significantly impact label naming or prescription advice in our trials, justifying the retention of the default setting for simplicity.
- The EasyOCR library features a limited number of tunable parameters, primarily concerning the text detection threshold and confidence level. We adopt the recommended default settings (e.g., a confidence threshold of 0.5) to ensure robust text extraction while minimizing the incidence of false positives.

## 4. Results

This section provides an in-depth analysis of the performance of the proposed Two-Stage OCR + LLM model and the Uni-Stage VQA model, evaluated using the RAGAs metrics framework [17,18,21]. The Two-Stage model integrates EasyOCR for text extraction and Qwen2-72b-instruct for language interpretation, while the Uni-Stage model employs Qwen2-72b-VL for direct multimodal processing of label images. The experimental evaluation focuses on the following RAGAs metrics: LLM Context Recall, Factual Correctness, Faithfulness, and Semantic Similarity and Standard classification metrics of accuracy, precision, recall, and F1-score.

### 4.1. Performance Comparison Based on RAGAs Metrics

Table 3 compares the performance of the Two-Stage and Uni-Stage models, highlighting the strengths and limitations of each approach. It should be noted that the Two-Stage model provides outputs that include label naming and prescription advice, making direct comparisons with state-of-the-art (SOTA) models, which primarily focus on label naming, inherently unequal. However, when accuracy is measured purely based on label naming, the Two-Stage model achieves 100% accuracy, surpassing all SOTA benchmarks.

### 4.2. Performance for Label Naming (Accuracy, Precision, Recall, F1-Score)

In addition to evaluating the models using RAGAs metrics, we also assess their performance specifically in *label naming*, treating it as a binary classification task. Out of our 100 prescription label images, each is either labeled correctly (i.e., the predicted drug name matches the ground truth) or incorrectly. Table 4 summarizes the results for both the Two-Stage and Uni-Stage models.

As seen in Table 4, the Two-Stage model (EasyOCR + Qwen2-72b-instruct) correctly labels all 100 samples, resulting in 100% accuracy. Consequently, precision, recall, and F1-score are all 1.00, confirming the model’s exceptional performance in identifying the correct drug name on prescription labels.

The Uni-Stage model (Qwen2-72b-VL) achieves a 96% accuracy, correctly labeling 96 out of 100 prescription images and misclassifying 4. Because there are no false positives in these 100 samples (it either labels an image correctly or fails outright), precision is 1.0000. However, recall drops to 0.9600, resulting in an overall F1-score of 0.9796.

These results reinforce the observation that the Two-Stage pipeline not only excels in context-rich interpretation (as evidenced by the RAGAs metrics) but also provides outstanding accuracy in basic label naming. Meanwhile, the Uni-Stage model offers a strong, albeit slightly lower, performance, balanced by faster inference speeds suitable for high-throughput scenarios.

### 4.3. Interpretation of Results

Table 3 highlights that the Two-Stage model outperforms the Uni-Stage model across all RAGAs metrics. Key insights are detailed below:

- LLM Context Recall: Scoring 0.88, the Two-Stage model demonstrates high retention and effective application of context from the drug label dataset, surpassing the Uni-Stage model’s score of 0.73. This indicates that sequential processing—first OCR, then LLM—enhances the model’s ability to retain context-rich information, which is essential for accurate interpretation of intricate medical instructions.
- Factual Correctness: Achieving a score of 0.83, the Two-Stage model benefits significantly from Retrieval-Augmented Generation (RAG), allowing it to validate extracted information against a curated pharmaceutical knowledge base. This enhances its reliability, particularly for accurately conveying dosage, administration instructions, and warnings. The Uni-Stage model, with a score of 0.69, demonstrates reasonable performance but shows limitations when more rigorous factual verification is required.
- Faithfulness: With a score of 0.76, the Two-Stage model adheres moderately well to the original label content, helping ensure accuracy in delivering medical information. The Uni-Stage model scored 0.64, suggesting it may occasionally introduce interpretive deviations, likely due to its single-step processing, which may simplify or generalize some details.
- Semantic Similarity: The Two-Stage model’s highest score of 0.91 in Semantic Similarity indicates strong alignment with expert-verified terminology and phrasing, which benefits elderly patients who require consistent and accurate instruction. The Uni-Stage model scored 0.78, showing it can provide reasonable clarity but could benefit from enhanced alignment with medical terminology to reduce ambiguity in its output.

### 4.4. Comparison with State-of-the-Art Models

To address the necessity for a comparative study with state-of-the-art (SOTA) models, we present a comparison of our Two-Stage model’s performance against leading drug label identification systems. Table 5 summarizes the accuracy of previous models and our Two-Stage model based on label naming accuracy.

**Table 5:** Comparison of label naming accuracy between our Two-Stage model and state-of-the-art models.

Study	Accuracy (%)	Notes
Liu et al. (2020) [22]	88.00	Multimodal integration; challenges with text complexity
Ting et al. (2020) [28]	96.26	Limitations with similar packaging
Gromova and Elangovan (2022) [29]	98.00	Focused on cylindrical bottles
You and Lin (2023) [33]	99.39	High precision on packaging types
Our Two-Stage Model	100.00	Includes label naming and prescription advice

As seen in Table 5, our Two-Stage model achieves a label naming accuracy of 100%, surpassing all previously reported accuracies in the literature. This exceptional performance highlights the effectiveness of our combined OCR and LLM approach in accurately identifying drug names from prescription labels.

It is important to note that while our model provides both label naming and prescription advice, which introduces additional complexity beyond simple label identification, we have focused the comparison on label naming accuracy to provide a fair assessment relative to previous models. The inclusion of prescription advice in our model offers added value for users, particularly elderly patients who benefit from comprehensive medication guidance.

The superior performance of our Two-Stage model can be attributed to several factors:

- EasyOCR demonstrates high accuracy in text extraction, effectively handling the diverse fonts and languages present in Thai prescription labels.
- The use of Qwen2-72b-instruct allows for nuanced interpretation of extracted text, improving the model’s ability to distinguish between similar drug names and handle complex label information.
- By integrating RAG with a curated drug dataset, the model cross-verifies label information, reducing errors and enhancing Factual Correctness.
- Our dataset includes a wide variety of prescription labels captured under realistic conditions, ensuring the model’s robustness and generalizability.

Compared to previous models, which often focus on specific packaging types or require extensive training on large datasets, our Two-Stage model offers a more adaptable and scalable solution. The ability to achieve 100% label naming accuracy without the need for extensive dataset-specific tuning represents a significant advancement in the field.

### 4.5. Comparative Analysis and Practical Implications

The inclusion of comparative results with SOTA models reinforces the superior performance of our Two-Stage model in drug label identification tasks. Given its consistently higher scores across RAGAs metrics and its 100% label naming accuracy, the Two-Stage model is ideally suited for tasks requiring detailed interpretation and strict adherence to medical standards. By leveraging sequential RAG-enhanced processing, it provides robust information verification, making it an effective tool for supporting medication guidance in settings where accuracy is paramount, such as elderly care consultations or home management of complex medication regimens.

In contrast, the Uni-Stage model, while not achieving the same level of accuracy and context retention, offers significant advantages in response speed and simplicity. This model’s streamlined design allows it to provide faster output, making it suitable for high-volume healthcare settings like pharmacies, where quick, general-purpose label interpretation is more critical than exhaustive detail. It effectively balances speed with accuracy, with some limitations in complex cases.

The RAGAs framework emphasizes the importance of accurate, contextually aligned, and faithful information in healthcare AI applications. The high scores in Factual Correctness and Semantic Similarity for the Two-Stage model highlight its suitability for precise medical tasks, particularly where maintaining fidelity to source information is critical. Conversely, the Uni-Stage model’s scores suggest it may be more effective for routine or repetitive tasks that benefit from rapid, albeit less detailed, responses.

Figure 2 and Figure 3 illustrate each model’s comparative performance and highlight their potential applications in healthcare settings:

- Two-Stage model (OCR + LLM): Given its consistently higher scores across RAGAs metrics, the Two-Stage model is ideally suited for tasks requiring detailed interpretation and strict adherence to medical standards. By leveraging sequential RAG-enhanced processing, it provides robust information verification, making it an effective tool for supporting medication guidance in settings where accuracy is paramount, such as elderly care consultations or home management of complex medication regimens.
- Uni-Stage model (VQA): While the Uni-Stage model does not achieve the same level of accuracy and context retention as the Two-Stage model, it offers significant advantages in response speed and simplicity. This model’s streamlined design allows it to provide faster output, making it suitable for high-volume healthcare settings like pharmacies, where quick, general-purpose label interpretation is more critical than exhaustive detail. It effectively balances speed with accuracy, albeit with some limitations in complex cases.
- Implications of RAGAs metrics: The RAGAs framework emphasizes the importance of accurate, contextually aligned, and faithful information in healthcare AI applications. The high scores in Factual Correctness and Semantic Similarity for the Two-Stage model highlight its suitability for precise medical tasks, particularly where maintaining fidelity to source information is critical. Conversely, the Uni-Stage model’s scores suggest it may be more effective for routine or repetitive tasks that benefit from rapid, albeit less detailed, responses.

### 4.6. Computational Complexity Analysis

We present a computational complexity analysis of both the Two-Stage (OCR + LLM) and Uni-Stage (VQA) models, evaluating their theoretical and practical computational demands. This examination encompasses the following:

- Time complexity: the theoretical scaling of processing time with respect to input size and model parameters.
- Memory footprint: GPU memory utilization during inference.
- Throughput: empirically measured inference speed under realistic operating conditions.

#### 4.6.1. Two-Stage Model: OCR + LLM

- OCR systems typically employ a convolutional backbone for feature extraction and a sequence decoder for text recognition. For an input image of size H×W, the convolutional feature extraction step exhibits a time complexity of O(H×W×k), where *k* represents the number of operations per convolutional kernel. The subsequent recurrent or transformer-based decoder has a complexity approximately proportional to O(L×d), with *L* being the length of the recognized text and *d* denoting the decoder’s hidden dimension.
- On an NVIDIA Tesla V100 GPU, EasyOCR processes a standard prescription label (under 1024×768 resolution) in approximately 70–100 ms per image. While increasing image resolution or text density extends inference time, it generally remains within feasible limits for real-time deployment in most outpatient settings.
- Modern transformer-based LLMs exhibit an inference complexity of $O(S^{2}\times D)$ per forward pass, where *S* is the sequence length (number of tokens) and *D* is the hidden dimension. Larger sequence lengths or model parameter counts lead to increased inference latency.
- For typical short prescription label text (ranging from 100 to 200 tokens), the average inference time is approximately 2000–3000 ms per query on a V100 GPU. The GPU memory footprint is typically 10–12 GB, varying slightly with batch size.

The total latency for the Two-Stage model, when chaining OCR and LLM inference, is approximately the sum of the latencies of the individual stages:

$$T_{2-\mathrm{Stage}}\approx T_{\mathrm{OCR}}+T_{\mathrm{LLM}}.$$

Empirical evaluations indicate a processing rate of approximately 2–3 s per image. While this throughput is lower compared to the Uni-Stage model, the Two-Stage approach consistently delivers superior Context Recall and Factual Correctness.

#### 4.6.2. Uni-Stage Model: VQA (Qwen2-72b-VL)

- A VQA system processes image features using a vision backbone (e.g., ViT or CNN). The image encoder’s complexity is roughly O(H×W×k), analogous to the OCR backbone stage. Subsequently, the combined vision–language transformer performs attention operations over both visual and textual tokens, resulting in a complexity of $O({\left(S_{v}+S_{t}\right)}^{2}\times D)$, where $S_{v}$ and $S_{t}$ represent the number of visual and textual tokens, respectively.
- As the Uni-Stage model integrates both vision and text processing into a single pass, it has a singular combined latency, typically ranging from 1800–2500 ms per image for our prescription label inputs. GPU memory usage often reaches 12–14 GB due to the concurrent encoding of visual and textual information.

By integrating OCR and language modeling into a unified step, the Uni-Stage approach achieves a processing rate of approximately 1.8–2.5 s per image under comparable batch settings on the same V100 GPU. This improved throughput presents an advantage in high-volume environments (e.g., pharmacy queues), albeit with a slight trade-off in Factual Correctness and Faithfulness compared to the Two-Stage model.

### 4.7. Summary of Findings and Recommendations

The analysis reveals distinct advantages for each model in different healthcare scenarios. The Two-Stage model, with its higher accuracy, context retention, and adherence to medical standards, is recommended for environments that require precise information and where the complexity of medication regimens demands thorough validation. This includes elderly patient consultations and contexts where medication adherence and safety are paramount.

In contrast, the Uni-Stage model, while less precise, offers a faster alternative for real-time applications, making it suitable for high-volume pharmacy settings and scenarios where quick access to basic information suffices. This model provides a viable option where rapid response is prioritized over exhaustive detail, such as in routine prescription labeling or quick reference scenarios.

## 5. Discussion

The findings of this study demonstrate the effectiveness of two distinct AI-driven models in interpreting prescription labels, providing essential support for elderly patients in managing their medications. Both the Two-Stage OCR + LLM model and the Uni-Stage VQA model showed strong potential in generating accessible, accurate, and contextually relevant interpretations of drug labels, each with distinct advantages suited to specific healthcare scenarios.

The Two-Stage model, integrating EasyOCR for text extraction [17] and Qwen2-72b-instruct for interpretation [18], consistently outperformed the Uni-Stage model across all RAGAs metrics [21]. With a notably higher LLM Context Recall score (0.88) and Semantic Similarity score (0.91), the Two-Stage model demonstrated a robust ability to retain context and align outputs with expert-verified terminology. These results suggest that the segmented OCR-to-LLM process enhances the model’s capability to produce context-rich, accurate responses that closely align with the complexities of medical information on drug labels. This is particularly advantageous for elderly patients who may require precise instructions and clear explanations to manage multiple medications.

When compared to state-of-the-art (SOTA) models such as Liu et al. (2020) [22] and You and Lin (2023) [33], the Two-Stage model demonstrates clear improvements in label naming accuracy, achieving 100% compared to 88.00% and 99.39%, respectively. This superior performance can be attributed to the integration of Retrieval-Augmented Generation (RAG) [21], enabling cross-referencing with a curated knowledge base like DrugBank for enhanced Factual Correctness. Moreover, unlike the SOTA models that focus primarily on specific packaging types or visual patterns, the Two-Stage model extends its functionality to include prescription advice, adding significant practical value for end-users.

The Uni-Stage VQA model, while not achieving the same level of accuracy or Faithfulness as the Two-Stage model, offers significant advantages in processing speed by interpreting visual and textual content in a single step. This streamlined approach makes it highly suitable for high-volume environments like pharmacies, where real-time response is critical. Its reasonable performance on RAGAs metrics, particularly in Semantic Similarity (0.78), highlights its potential for routine applications where quick, general-purpose label interpretation suffices [39].

Evaluating these models based on the RAGAs framework [21] highlights the importance of balancing accuracy and processing speed depending on the specific healthcare context. The Two-Stage model’s high scores in Factual Correctness (0.83) and Faithfulness (0.76) underscore its utility in scenarios that require strict adherence to the original information, making it ideal for use in settings where a misinterpretation could lead to significant health risks. Conversely, the Uni-Stage model’s relatively lower yet reasonable scores in these areas (0.69 for Factual Correctness and 0.64 for Faithfulness) suggest its suitability for scenarios where general guidance rather than precise detail is acceptable.

Despite these positive findings, the proposed models have limitations. One significant challenge lies in their reliance on OCR quality for text extraction. Labels with curved surfaces, low contrast, or unconventional fonts can impact accuracy, propagating errors through the pipeline, especially in the Two-Stage model. Addressing these challenges will require further development of OCR technology and multimodal approaches to enhance robustness in diverse real-world scenarios.

## 6. Conclusions and Future Work

In conclusion, this study confirms the effectiveness of AI-driven systems for enhancing drug label interpretation and medication management, offering valuable insights into the unique strengths of two distinct model architectures. By integrating Optical Character Recognition, Large Language Models, and Retrieval-Augmented Generation, the Two-Stage and Uni-Stage models provide elderly patients and healthcare providers with accessible, accurate, and contextually tailored drug information. Notably, the Two-Stage model achieves a 100% label naming accuracy, surpassing state-of-the-art benchmarks, while the Uni-Stage model offers rapid interpretation suitable for high-throughput environments like pharmacies. These findings highlight the potential for AI-assisted solutions to bridge critical gaps in medication management, particularly for elderly populations.

Future work will expand this system into a fully multimodal pipeline that integrates additional input and output capabilities, enhancing usability and accessibility for elderly users. By incorporating Automatic Speech Recognition (ASR) technologies like Whisper [53], the system could support spoken queries, allowing patients to obtain drug information through voice commands—a feature that is particularly beneficial for individuals with reduced vision or mobility. Coupling ASR with Text-to-Speech (TTS) functionality would enable the system to deliver audible responses, transforming the interaction experience for patients who face barriers to reading or handling mobile devices. These innovations could significantly enhance accessibility, making AI support feasible for a broader range of users.

To further improve personalization, future iterations should incorporate patient-specific information, such as individual medication histories and potential contraindications. Secure integration with electronic health records (EHRs) could enable the system to adjust recommendations and warnings based on a patient’s unique health profile, ensuring safer and more tailored medication management.

While this study utilized a dataset of 100 prescription label images, broader data collection efforts are essential for capturing the diversity of real-world scenarios. Expanding the dataset to include various medications, label formats, languages, and lighting conditions will improve the models’ generalizability and robustness. Additionally, periodic retraining will ensure the models remain up-to-date with evolving pharmaceutical regulations, label designs, and newly introduced medications.

Finally, longitudinal studies on user engagement and medication adherence are necessary to understand the broader impact of these AI tools on health outcomes. Such studies will provide critical insights into their effectiveness in improving medication management, particularly among elderly populations, and guide refinements to optimize their interaction strategies for clinical and home healthcare applications.

## Figures and Tables

**Table 2:** Overview of medication label data collection.

Drug Group	Unique Drug Names	Dosage Information
Pain Relief	Naproxen	250 mg
	Paracetamol	500 mg
	Tramadol HCl	50 mg
	Aspirin	81 mg
	NorGesic (Paracetamol + Orphrenadrine)	500 + 35 mg
Cold Relief	Glyceryl Guaicolate	100 mg
	Dextromethorphan	15 mg
	Acetylcysteine	200 mg
Vitamins	Folic Acid	5 mg
	B-Co Vitamin	-
	Multivitamins	-
	Vitamin B6	50 mg
Blood Pressure	Amlodipine	10 mg
	Enalapril	5 mg
	Losartan	50 mg
	Manidipine	20 mg
	Hydralazine HCL	25 mg
Cholesterol	Simvastatin	10 mg, 40 mg
	Gemfibrozil	300 mg
Diabetes	Metformin	500 mg
	Glipizide	5 mg
Antibiotics	Ciprofloxacin	250 mg
	Amoxicillin	500 mg
Gastrointestinal	Omeprazole	20 mg
	Domperidone	10 mg
	Sodium Bicarbonate	300 mg
	Calcium Carbonate	600 mg
Mental Health	Lorazepam	1 mg
	Risperidone	2 mg
	Amitriptyline	10 mg
	Sertraline	50 mg
	Fluoxetine	20 mg
	Chlorpromazine	50 mg
	Benzhexol HCL	2 mg
	Gabapentin	300 mg
Hormones	Progesterone	200 mg
	Calcitriol (Vitamin D3)	0.25 mcg
	Ergocalciferol (Vitamin D2)	20,000 IU
Iron Supplements	Ferrous Fumarate	200 mg
Miscellaneous	SIMEthicone	80 mg
	Milk of Magnesia	-
	Acetylcysteine	-
	Rifampicin	300 mg
	Isoniazid	100 mg
	Pantoprazole	20 mg
	Natear (Hypromellose + Boric Acid)	-
	ORS	7.5 g

**Table 3:** Performance comparison of Two-Stage (EasyOCR + Qwen2-72b-instruct) and Uni-Stage (Qwen2-72b-VL) models based on RAGAs metrics.

Metric	Two-Stage Model	Std Dev (Two-Stage)	Uni-Stage Model	Std Dev (Uni-Stage)
LLM Context Recall	0.88	±0.04	0.73	±0.05
Factual Correctness	0.83	±0.05	0.69	±0.06
Faithfulness	0.76	±0.06	0.64	±0.07
Semantic Similarity	0.91	±0.03	0.78	±0.04

**Table 4:** Label naming results for Two-Stage and Uni-Stage models (binary classification on 100 samples).

Model	#Correct	#Incorrect	Accuracy	Precision	Recall	F1-Score
Two-Stage	100	0	100.00%	1.0000	1.0000	1.0000
Uni-Stage	96	4	96.00%	1.0000	0.9600	0.9796

## Data Availability

Data are contained within the article.

## References

[1] Department of Older Persons, Ministry of Social Development and Human Security Situation of the Thai Older Persons 2021. https://www.dop.go.th/download/knowledge/th1663828576-1747_1.pdf.

[2] Department of Older Persons Statistics of Older Persons June 2023 by Looker Studio. https://www.dop.go.th/th/know/side/1/1/2449.

[3] Economic Research Institute for ASEAN and East Asia (ERIA) Population Ageing in Thailand. https://www.eria.org/publications/population-ageing-in-thailand.

[4] World Health Organization Thailand’s Leadership and Innovations Towards Healthy Ageing. https://www.who.int/southeastasia/news/feature-stories/detail/thailands-leadership-and-innovation-towards-healthy-ageing.

[5] Vinks T.H., De Koning F.H., de Lange T.M., Egberts T.C. (2006). Identification of Potential Drug-Related Problems in the Elderly: The Role of the Community Pharmacist. Pharm. World Sci..

[6] Sapkota S., Pudasaini N., Singh C., Sagar G.C. (2011). Drug Prescribing Pattern and Prescription Error in Elderly: A Retrospective Study of Inpatient Record. Asian J. Pharm. Clin. Res..

[7] Yang C., Zhu S., Lee D.T.F., Chair S.Y. (2022). Interventions for Improving Medication Adherence in Community-Dwelling Older People with Multimorbidity: A Systematic Review and Meta-Analysis. Int. J. Nurs. Stud..

[8] Stock S., Redaelli M., Simic D., Siegel M., Henschel F. (2014). Risk Factors for the Prescription of Potentially Inappropriate Medication (PIM) in the Elderly. Wien. Klin. Wochenschr..

[9] Roux-Marson C., Baranski J.B., Fafin C., Exterman G., Vigneau C., Couchoud C., Moranne O. (2020). Medication Burden and Inappropriate Prescription Risk Among Elderly with Advanced Chronic Kidney Disease. BMC Geriatr..

[10] Noonan M.C., Wingham J., Taylor R.S. (2018). Who Cares? The Experiences of Caregivers of Adults Living with Heart Failure, Chronic Obstructive Pulmonary Disease and Coronary Artery Disease: A Mixed Methods Systematic Review. BMJ Open.

[11] Khanagar S.B., Al-Ehaideb A., Maganur P.C., Vishwanathaiah S., Patil S., Baeshen H.A., Sarode S.C., Bhandi S. (2021). Developments, Application, and Performance of Artificial Intelligence in Dentistry—A Systematic Review. J. Dent. Sci..

[12] Loh H.W., Ooi C.P., Seoni S., Barua P.D., Molinari F., Acharya U.R. (2022). Application of Explainable Artificial Intelligence for Healthcare: A Systematic Review of the Last Decade (2011–2022). Comput. Methods Programs Biomed..

[13] Yin J., Ngiam K.Y., Teo H.H. (2021). Role of Artificial Intelligence Applications in Real-Life Clinical Practice: Systematic Review. J. Med. Internet Res..

[14] Albahri O.S., Zaidan A.A., Albahri A.S., Zaidan B.B., Abdulkareem K.H., Al-Qaysi Z.T., Alamoodi A.H., Aleesa A.M., Chyad M.A., Alesa R.M. (2020). Systematic Review of Artificial Intelligence Techniques in the Detection and Classification of COVID-19 Medical Images in Terms of Evaluation and Benchmarking: Taxonomy Analysis, Challenges, Future Solutions and Methodological Aspects. J. Infect. Public Health.

[15] Goodfellow I., Bengio Y., Courville A. (2016). Deep Learning.

[16] He K., Zhang X., Ren S., Sun J. Deep Residual Learning for Image Recognition. Proceedings of the IEEE Conference on Computer Vision and Pattern Recognition.

[17] Jaided AI, EasyOCR. https://github.com/JaidedAI/EasyOCR.

[18] Yang A., Yang B., Hui B., Zheng B., Yu B., Zhou C., Fan Z. (2024). Qwen2 Technical Report. arXiv.

[19] Lewis P., Oguz B., Rinott R., Riedel S., Stoyanov V. Retrieval-Augmented Generation for Knowledge-Intensive NLP Tasks. Proceedings of the 34th International Conference on Neural Information Processing Systems.

[20] Wishart D.S., Feunang Y.D., Guo A.C., Lo E.J., Marcu A., Grant J.R., Sajed T., Johnson D., Li C., Sayeeda Z. (2018). DrugBank 5.0: A Major Update DrugBank Database 2018. Nucleic Acids Res..

[21] Es S., James J., Espinosa-Anke L., Schockaert S. (2023). RAGAs: Automated Evaluation of Retrieval Augmented Generation. arXiv.

[22] Liu X., Meehan J., Tong W., Wu L., Xu X., Xu J. (2020). DLI-IT: A Deep Learning Approach to Drug Label Identification through Image and Text Embedding. BMC Med. Inform. Decis. Mak..

[23] Tian Z., Huang W., He T., He P., Qiao Y. Detecting Text in Natural Image with Connectionist Text Proposal Network. Proceedings of the European Conference on Computer Vision.

[24] Simonyan K., Zisserman A. (2014). Very Deep Convolutional Networks for Large-Scale Image Recognition. arXiv.

[25] Hochreiter S., Schmidhuber J. (1997). Long Short-Term Memory. Neural Comput..

[26] Smith R. An Overview of the Tesseract OCR Engine. Proceedings of the Ninth International Conference on Document Analysis and Recognition (ICDAR 2007).

[27] Redmon J., Farhadi A. (2016). YOLO9000: Better, Faster, Stronger. arXiv.

[28] Ting H.W., Chung S.L., Chen C.F., Chiu H.Y., Hsieh Y.W. (2020). A Drug Identification Model Developed Using Deep Learning Technologies: Experience of a Medical Center in Taiwan. BMC Health Serv. Res..

[29] Gromova K., Elangovan V. (2022). Automatic Extraction of Medication Information from Cylindrically Distorted Pill Bottle Labels. Mach. Learn. Knowl. Extract..

[30] Szeliski R. (2022). Computer Vision: Algorithms and Applications.

[31] Tan M., Le Q.V. EfficientNet: Rethinking Model Scaling for Convolutional Neural Networks. Proceedings of the 36th International Conference on Machine Learning.

[32] Howard A.G., Zhu M., Chen B., Kalenichenko D., Wang W., Weyand T., Andreetto M., Adam H. (2017). MobileNets: Efficient Convolutional Neural Networks for Mobile Vision Applications. arXiv.

[33] You Y.S., Lin Y.S. (2023). A Novel Two-Stage Induced Deep Learning System for Classifying Similar Drugs with Diverse Packaging. Sensors.

[34] Huang S.C., Pareek A., Seyyedi S., Banerjee I., Lungren M.P. (2020). Fusion of Medical Imaging and Electronic Health Records Using Deep Learning: A Systematic Review and Implementation Guidelines. NPJ Digit. Med..

[35] Kumar Y., Koul A., Singla R., Ijaz M.F. (2023). Artificial Intelligence in Disease Diagnosis: A Systematic Literature Review, Synthesizing Framework and Future Research Agenda. J. Ambient Intell. Humaniz. Comput..

[36] Hu S., Yu S., Cheng C., Shen X., Zhang H., Wei F., Zhou X., Yu H., Zhuang L., Liu J. MiniCPM: Unveiling the Potential of Small Language Models with Scalable Training Strategies. https://arxiv.org/abs/2404.06395.

[37] Zhao W.X., Zhou K., Li J., Tang T., Wang X., Hou Y., Min Y., Zhang B., Zhang J., Dong Z. (2023). A Survey of Large Language Models. arXiv.

[38] Harris J., Laurence T., Loman L., Grayson F., Nonnenmacher T., Long H., WalsGriffith L., Douglas A., Fountain H., Georgiou S. (2024). Evaluating Large Language Models for Public Health Classification and Extraction Tasks. arXiv.

[39] Awais M., Naseer M., Khan S., Anwer R.M., Cholakkal H., Shah M., Khan F.S. (2023). Foundational Models Defining a New Era in Vision: A Survey and Outlook. arXiv.

[40] Hartsock I., Rasool G. (2024). Vision-Language Models for Medical Report Generation and Visual Question Answering: A Review. arXiv.

[41] Conneau A., Khandelwal K., Goyal N., Chaudhary V., Wenzek G., Guzman F., Stoyanov V. Unsupervised Cross-Lingual Representation Learning at Scale. Proceedings of the 58th Annual Meeting of the Association for Computational Linguistics.

[42] Xue L., Constant N., Roberts A., Kale M., Al-Rfou R., Siddhant A., Barua A., Raffel C. (2021). mT5: A Massively Multilingual Pre-Trained Text-to-Text Transformer. arXiv.

[43] Anil R., Dai A.M., Firat O., Johnson M., Lepikhin D., Passos A., Wu Y. (2023). PaLM 2 Technical Report. arXiv.

[44] Jiang A.Q., Sablayrolles A., Mensch A., Bamford C., Chaplot D.S., Casas D.D.L., Bressand F., Lengyel G., Lample G., Saulnier L. (2023). Mistral 7B. arXiv.

[45] Laurençon H., Saulnier L., Wang T., Akiki C., Villanova del Moral A., Le Scao T., Jernite Y. (2022). The BigScience Roots Corpus: A 1.6 TB Composite Multilingual Dataset. Adv. Neural Inf. Process. Syst..

[46] OpenAI ChatGPT (July 5 Version) [Large Language Model]. https://chat.openai.com/.

[47] Gu Y., Tinn R., Cheng H., Lucas M., Usuyama N., Liu X., Poon H. (2021). Domain-Specific Language Model Pretraining for Biomedical Natural Language Processing. ACM Trans. Comput. Healthc..

[48] Singhal K., Azizi S., Tu T., Mahdavi S.S., Wei J., Chung H.W., Natarajan V. (2023). Large Language Models Encode Clinical Knowledge. Nature.

[49] Johnson J., Hariharan B., Van Der Maaten L., Fei-Fei L., Lawrence Zitnick C., Girshick R. CLEVR: A Diagnostic Dataset for Compositional Language and Elementary Visual Reasoning. Proceedings of the IEEE Conference on Computer Vision and Pattern Recognition.

[50] Hudson D.A., Manning C.D. GQA: A New Dataset for Real-World Visual Reasoning and Compositional Question Answering. Proceedings of the IEEE/CVF Conference on Computer Vision and Pattern Recognition.

[51] Lau J.J., Gayen S., Ben Abacha A., Demner-Fushman D. (2018). A Dataset of Clinically Generated Visual Questions and Answers About Radiology Images. Sci. Data.

[52] He X., Zhang Y., Mou L., Xing E., Xie P. (2020). PathVQA: 30000+ Questions for Medical Visual Question Answering. arXiv.

[53] Radford A., Kim J.W., Xu T., Brockman G., McLeavey C., Sutskever I. (2022). Robust Speech Recognition via Large-Scale Weak Supervision. arXiv.

